# Polymodal TRPV1 and TRPV4 Sensors Colocalize but Do Not Functionally Interact in a Subpopulation of Mouse Retinal Ganglion Cells

**DOI:** 10.3389/fncel.2018.00353

**Published:** 2018-10-16

**Authors:** Monika Lakk, Derek Young, Jackson M. Baumann, Andrew O. Jo, Hongzhen Hu, David Križaj

**Affiliations:** ^1^Department of Ophthalmology and Visual Sciences, University of Utah, Salt Lake City, UT, United States; ^2^Department of Bioengineering, University of Utah, Salt Lake City, UT, United States; ^3^Department of Anesthesiology, Washington University School of Medicine, St. Louis, MO, United States; ^4^Department of Neurobiology and Anatomy, University of Utah, Salt Lake City, UT, United States

**Keywords:** retina, calcium, TRPV1, TRPV4, endocannabinoids, glaucoma, RGC

## Abstract

Retinal ganglion cells (RGCs) are projection neurons that transmit the visual signal from the retina to the brain. Their excitability and survival can be strongly influenced by mechanical stressors, temperature, lipid metabolites, and inflammatory mediators but the transduction mechanisms for these non-synaptic sensory inputs are not well characterized. Here, we investigate the distribution, functional expression, and localization of two polymodal transducers of mechanical, lipid, and inflammatory signals, TRPV1 and TRPV4 cation channels, in mouse RGCs. The most abundant vanilloid mRNA species was *Trpv4*, followed by *Trpv2* and residual expression of *Trpv3* and *Trpv1*. Immunohistochemical and functional analyses showed that TRPV1 and TRPV4 channels are expressed as separate molecular entities, with TRPV1-only (∼10%), TRPV4-only (∼40%), and TRPV1 + TRPV4 (∼10%) expressing RGC subpopulations. The TRPV1 + TRPV4 cohort included SMI-32-immunopositive alpha RGCs, suggesting potential roles for polymodal signal transduction in modulation of fast visual signaling. Arguing against obligatory heteromerization, optical imaging showed that activation and desensitization of TRPV1 and TRPV4 responses evoked by capsaicin and GSK1016790A are independent of each other. Overall, these data predict that RGC subpopulations will be differentially sensitive to mechanical and inflammatory stressors.

## Introduction

Vertebrate vision is based on separating photon input from background thermal energy and extraction of luminance, local contrast, color, orientation, direction of motion, and “looming” information from the visual scene (Lettvin et al., 1959). Feature extraction is conducted in parallel by over 40 types of retinal ganglion cells (RGCs), which project axons from the retina to a wide range of midbrain nuclei (Morin and Studholme, 2014; Baden et al., 2016). RGCs are typically categorized by their light response and serendipitous expression of molecular markers (Zeng and Sanes, 2017) but this ignores the possibility that RGC might also be classified based on their responsiveness to the local milieu, which continually bombards them with mechanical, cardiovascular and immune signals. We know that non-canonical non-synaptic sensory inputs can dramatically impact the function and survival of RGC subtypes (Muller et al., 2014; Duan et al., 2015; Križaj, 2016; Ou et al., 2016) yet the lack of knowledge about the transduction mechanisms that mediate them hampers physiological insight and treatment in diseases such as glaucoma, diabetic retinopathy, ischemia, and traumatic ocular injury.

Transient receptor potential (TRP) vanilloid channels are polymodal cation channels that function as molecular integrators of many types of sensory input (Clapham, 2003; Nilius and Szallasi, 2014). The 28 isoforms that constitute the TRP superfamily function as transducers of the ambient physico-chemical and inflammatory environment due to their sensitivity for mechanical stressors (strain, pressure, shear flow, swelling), temperature, pH, lipid, and inflammatory metabolites. Because the channels are permeable to Ca^2+^ and can be activated at resting membrane potentials, they are able to modulate neuronal physiology in the absence of synaptic activation (Bradshaw et al., 2013; Redmon et al., 2017). An archetypal example is the dorsal root ganglion (DRG), composed of sensory neuron populations that can be classified by the relative expression of TRPV1 nociceptors, TRPV4 osmosensors, TRPM8 innocuous pain sensors, TRPA1 cold pain sensors, TRPM2 redox sensors, with TRPC1, TRPC6, and TRPV2 channels adding additional layers of complexity (Sousa-Valente et al., 2014; Teichert et al., 2014). Within the vanilloid subfamily, TRPV1-4 (also known as thermoTRPs for their temperature sensitivity) are non-selective cation channels whereas TRPV5 and TRPV6 are predominantly permeable to Ca^2+^ and typically expressed in epithelial and bone cells (Clapham, 2003). The most studied vanilloid isoforms are TRPV1 and TRPV4, with ∼50% sequence homology and activation by distinct agonists, temperature ranges, mechanical, osmotic, and inflammatory stressors (Martins et al., 2014; Nilius and Szallasi, 2014). Gene association and clinical studies identified single-nucleotide polymorphisms in the coding/promoter regions of TRPV4 with mutations that cause debilitating sensory and motor neuropathies and musculoskeletal disorders (Nilius and Voets, 2013; Echaniz-Laguna et al., 2014). Inflammatory agents sensitize TRPV1/4 channels by mechanisms that are not fully defined whereas selective inhibition or deletion of TRPV1- and TRPV4-expressing neurons produces burning, freezing, itch, mechanical pain, and thermosensory phenotypes together with loss of osmoregulation and hearing loss (Caterina et al., 1997; Tominaga et al., 1998; Liedtke and Friedman, 2003; Alessandri-Haber et al., 2004).

Vertebrate retinas express many – perhaps most – TRP isoforms (Gilliam and Wensel, 2011; Križaj, 2016) yet studies of TRP signaling are in their infancy and it is unknown whether different isoforms work together to transduce complementary features of the intra-retinal milieu. In contrast to the canonical TRPC1 isoform which is expressed in most retinal cells (Molnar et al., 2012, 2016), the most extensively studied isoform – TRPV1 – was localized to photoreceptors and subsets of RGCs, bipolar, and amacrine cells (Yazulla, 2008; Middleton and Protti, 2011; Ryskamp et al., 2014a; Jo et al., 2017). Its cognate, TRPV4, has been detected in RGCs, Müller glia, and endothelial cells (Ryskamp et al., 2011, 2014b; Jo et al., 2015; Phuong et al., 2017; Taylor et al., 2017) but, unlike TRPV1, appears to be absent from amacrine, bipolar, and photoreceptor cells (Yarishkin et al., 2018). The relative expression of vanilloid isoforms across RGCs is unknown, nor is it clear whether TRPV1 and TRPV4 colocalize and/or can interact. Because heteromultimerization could increase the cells’ capacity to sense changes in ambient environment, we studied the relative expression levels of TRPV1 and TRPV4 channels, investigated their functional distribution and integration, and tested the influence of channel activation on cellular calcium homeostasis in mouse RGCs. We identified distinct RGC constellations that include TRPV4-, TRPV1-, and TRPV1 + TRPV4-expressing populations in which TRPV1 and TRPV4 channels are activated independently. These data suggest that sensing of ambient information (temperature, mechanical stress, pH, and endocannabinoids) across physiological and pathological ranges may be differentially distributed across RGC populations.

## Materials and Methods

### Ethical Approval and Animals

Animal handling, anesthetic procedures, and experiments were performed in accordance with the NIH Guide for the Care and Use of Laboratory Animals and the ARVO Statement for the Use of Animals in Ophthalmic and Vision Research. The project was approved by the Institutional Animal Care and Use Committees at the University of Utah. We assessed retinal TRPV1 expression using a knock-in mouse in which *Cre* was inserted into Exon 15 of *Trpv1* (TRPV1Cre; Jackson Laboratory 017769). This line was crossed to B6.Cg-*Gt(ROSA)26Sortm9(CAG-tdTomato)Hze*/J (Ai9; 007909) in which the LoxP-STOP-LoxP TdTomato construct is knocked in at the *Gt(ROSA)26Sor* locus (Madison et al., 2010; Jo et al., 2017). *Trpv4*^-/-^ mice have an excised exon 12-encoding transmembrane pore domains 5 and 6 (Liedtke and Friedman, 2003). C57BL/6J (C57), bacterial artificial chromosome (BAC)-transgenic Tg(TRPV4- EGFP)MT43Gsat mice (referred to as TRPV4^eGFP^), TRPV1^-/-^, TRPV4^-/-^, *TRPV1Cre:Ai3*, and *Trpv1Cre:Ai9* mice were maintained in a pathogen-free facility with a 12-h light/dark cycle and unrestrained access to food and water. Data were gathered from male and female mice with no noted gender differences.

### Reagents

The TRPV4 agonist GSK1016790A (GSK101) and antagonist HC-067047 (HC-06) were purchased from Sigma. The TRPV1 agonist capsaicin (CAP; 8-methyl-*N*-vanillyl-6-nonenamide) and the TRPV1 antagonist capsazepine (CPZ; *N*-[2-(4-Chlorophenyl)ethyl]-1,3,4,5-tetrahydro-7,8-dihydroxy-2*H*-2-benzazepine-2-carbothioamide) and the endogenous agonist of CB1 receptors 2-arachidonoylglycerol (2-AG) were obtained from Cayman Chemicals. BDNF and CNTF used to culture RGCs were obtained from GenWay Biotech. Other salts and reagents were purchased from Sigma, VWR, Across Organics, or Thermo Fisher. GSK101 (1 mM), HC-06 (10 mM), CAP (10 mM), and CPZ (20 mM) stocks in DMSO were diluted in extracellular saline before use and placed into reservoirs connected to gravity-fed perfusion systems (Warner Instruments).

### Magnetic-Activated Cell Sorting (MACS)

The retinas were incubated in an enzyme solution containing 16 U/ml papain (Worthington), 0.2 mg/ml L-cysteine (Sigma), and 50 U/ml DNase I recombinant (Roche) for 45 min at 37°C with gentle agitation and triturated with D-PBS solution containing 1.5 mg/ml BSA, 1.5 mg/ml Trypsin inhibitor, pH: 7.4, to yield a single cell suspension that was passed through a 30 μm pre-separation filter and centrifuged. The cell pellet was re-suspended and incubated in 0.5% BSA solution containing CD90.1 MicroBeads (1:10; Miltenyi Biotech) for 15 min at 4°C. After additional washing and centrifugation, cells were separated using MACS LS columns and incubated in serum-free neurobasal medium (Gibco/ThermoFisher) with 1% penicillin/streptomycin (Sciencell), transferrin (0.1 mg/ml), putrescine (16 ng/ml), insulin (5 μg/ml), 3,5,3-triiodothyronine T3 (100 nM), progesterone (20 nM), 2% B27, N-acetyl cysteine (5 ng/ml), sodium pyruvate (1 mM), L-glutamine (2 mM), brain-derived neurotrophic factor (BDNF, 50 ng/ml), ciliary neurotrophic factor (CNTF, 10 ng/ml), and forskolin (5 μM). The growth medium was changed every 2–3 days.

### Semiquantitative Real-Time PCR

Total RNA was isolated using the Arcturus PicoPure RNA Isolation Kit (Applied Biosystems) as described (Phuong et al., 2017). One microgram of total RNA was used for reverse transcription. First-strand cDNA synthesis and PCR amplification of cDNA were performed using qScript^TM^ XLT cDNA SuperMix cDNA synthesis kit (Quanta Biosciences). The PCR products were run on 2% agarose gels and visualized by ethidium bromide staining, along with 100-bp DNA ladder (ThermoFisher). SYBR Green-based real-time PCR was performed using Apex qPCR Master Mix (Genesee Scientific). The results were performed in triplicate of at least four separate experiments. The comparative C_T_ method (ΔΔC_T_) was used to measure relative gene expression where the fold enrichment was calculated as: 2^-[ΔCT(sample)-ΔCT(calibrator)]^ after normalization. To normalize fluorescence signals, GAPDH and β-actin were utilized as endogenous controls. The primer sequences and expected product sizes are given in **Table 1**.

**Table 1:** Primer sequences used for PCR and semiquantitative real-time PCR analysis.

Name	Forward primer	Reverse primer	Product size (bp)
*Trpv1*	AGGGTGGATGAGGTGAACTGGACT	GCTGGGTGCTATGCCTATCTCG	199
*Trpv2*	GTTGGCCTACGTCCTCCTCACCTA	TGCACCACCAGTAACCATTCTCC	158
*Trpv3*	CTCACCTTCGTCCTCCTCCTCAAC	CAGCCGGAAGTCCTCATCTGCTA	201
*Trpv4*	TCCTGAGGCCGAGAAGTACA	TCCCCCTCAAACAGATTGGC	166
*Gapdh*	GGTTGTCTCCTGCGACTTCA	TAGGGCCTCTCTTCCTCAGT	220
*Actb*	CCACCATGTACCCAGGCATT	AGGGTGTAAAACGCAGCTCA	253


### Retinal Dissociation and Optical Imaging

The animals were euthanized by isoflurane inhalation. The retinas were isolated in ice-cold Leibovitz 15 (L15) medium containing 11 mg/ml L15 powder, with (in mM) 20 D-glucose, 10 Na-HEPES, 2 sodium pyruvate, 0.3 sodium ascorbate, and 1 glutathione. Incubation in L15 containing papain (7 U/ml; Worthington) digested the extracellular matrix, and was terminated by rinsing with cold L15 solution; 500 μm pieces of retina were mechanically dissociated and cells were plated onto concanavalin A (0.2–0.5 mg/ml) coated coverslips. Calcium imaging followed established protocols (Ryskamp et al., 2011; Jo et al., 2016; Lakk et al., 2017), with the cells loaded with the Fura-2 AM (5–10 μM, Life Technologies) indicator dye for 45–60 min. Extracellular saline contained: (in mM) 133 NaCl, 10 HEPES hemisodium salt, 10 glucose, 2.5 KCl, 2 CaCl_2_, 1.5 MgCl_2_, 1.25 NaH_2_PO_4_, 1 pyruvic acid, 1 lactic acid, and 0.5 glutathione. Epifluorescence images were acquired using an inverted Nikon microscope with a 40x (1.3 NA oil) objective. Subsets of cells were stimulated with agonists and antagonists of TRPV1/4 channels. 340 and 380 nm excitation was delivered from an Xe lamp (Lambda DG-4, Sutter Instruments). Emissions were collected at 510 nm with 14-bit CoolSNAPHQ2 or Delta Evolve cameras and analyzed using NIS-Elements. Δ*R*/*R* (peak *F*340/*F*380 ratio – baseline/baseline) was used to quantify the amplitude of Ca^2+^ signals.

### Immunofluorescence

The immunolabeling protocol for vertical sections followed the procedures described in Molnar et al. (2016) and Jo et al. (2017). The retinas were fixed for 1 h in 4% paraformaldehyde, rinsed with PBS, dehydrated, and embedded in OCT compound mounting medium (Electron Microscopy Sciences); 12 μm thick cryosections were incubated in a blocking buffer (5% FBS and 0.3% Triton X-100 in 1X PBS) for 20 min. Primary antibodies (rabbit anti-TRPV4, 1:1000, LifeSpan Biosciences; rabbit anti-RBPMS, 1:500, PhosphoSolutions; mouse anti-Thy1.1, Sigma, 1:500; mouse SMI-32, 1:100, Covance; mouse anti-GFP, 1:500, Santa Cruz) were diluted in the diluent (2% BSA and 0.2% Triton X-100 in 1X PBS) and applied overnight at 4°C, followed by incubation in fluorophore-conjugated secondary antibodies (1:500; goat anti-mouse AlexaFluor 405, 488, or 647, goat anti-rabbit AlexaFuor 488 or 594, Life Technologies) for 1 h at RT. Images were acquired on an Olympus CV1200 confocal microscope using 20x (NA water) and 40x (0.9 NA water) objectives.

### Statistical Analysis

Data are presented as means ± SEM. Statistical comparisons were made with one-way ANOVA test followed by *post hoc* Tukey’s multiple comparison of means (Origin 8.0, Origin Lab Corporation). A difference of *P* ≤ 0.05 (^∗^), *P* ≤ 0.01 (^∗∗^), *P* ≤ 0.001 (^∗∗∗^), and *P* ≤ 0.0001 (^∗∗∗∗^) were considered statistically significant.

## Results

### Mammalian RGCs Express Multiple Vanilloid TRP Isoforms

**FIGURE 1:**
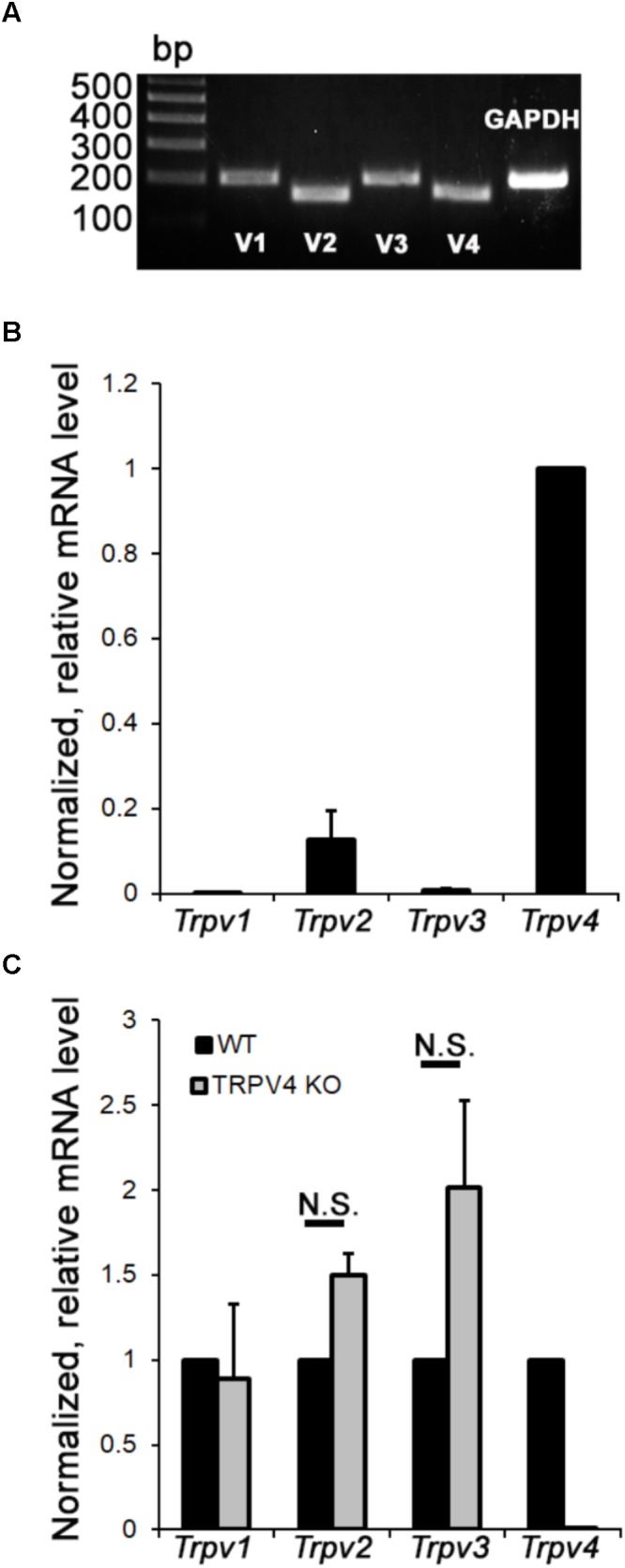
Relative mRNA expression levels of vanilloid thermoTRP channels in primary mouse RGCs. **(A)** End point PCR. RGCs express all four thermo *Trpv* transcripts. *Gapdh* (glyceraldehyde 3-phosphate dehydrogenase) mRNA served as a loading control. **(B)** Semiquantitative RT-PCR. The relative abundance of thermo *Trpv* transcripts normalized with respect to *Trpv4* content (*n* = 4). **(C)** Fold change in mRNA expression in TRPV4*^-^*^/^*^-^* RGCs (*n* = 4) was calculated relative to expression in wild type cells.

Vanilloid TRP channels are osmo- and thermosensitive non-selective cation channels with critical functions in neuronal neural plasticity, synaptic transmission, synapse formation, neurogenesis, apoptosis, and survival (Sousa-Valente et al., 2014; Ramírez-Barrantes et al., 2016; White et al., 2016). The mouse retina expresses multiple members of the vanilloid subfamily (Gilliam and Wensel, 2011) but their relative expression in RGCs is not known. RNA profiling shows that mouse RGCs express all four thermo*Trp* transcripts (*Trpv1-4*) (**Figure 1A**), with expression dominated by *Trpv4*, followed by *Trpv2*, *Trpv3*, and *Trpv1* mRNAs, respectively (**Figure 1B**). TRPV4^-/-^ RGCs showed a trend toward *Trpv1* upregulation but these changes were not significant (**Figure 1C**). Examination of mRNAs in TRPV4^-/-^ RGCs showed little evidence of cross-isoform plasticity apart from a (non-significant) trend toward *Trpv1* (**Figure 1C**). We conclude that mouse RGCs as a group express all non-epithelial vanilloid *Trp* genes. Lack of compensatory upregulation in KO mice lacking the dominant TRPV4 channel might indicate absence of regulatory interaction at the transcriptional level, or an absence of obligatory heteromerization.

### Pharmacological Activation of TRPV1 and TRPV4 Channels Reveals Functional Overlap in a Subset of RGCs

**FIGURE 2:**
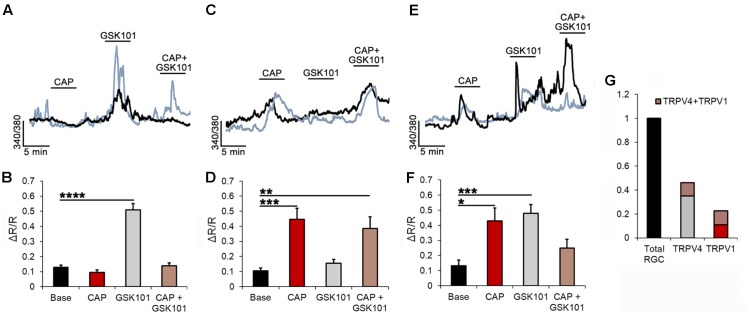
TRPV1 and TRPV4 modulate Ca^2+^ homeostasis in subsets of RGCs. **(A)** Two simultaneously recorded RGCs (denoted by black and blue traces) respond to GSK101 with [Ca^2+^]_i_ elevations but are insensitive to CAP. **(B)** Averaged data for the GSK101-responding CAP-insensitive pool of cells (*n* = 87). **(C)** Representative traces of CAP responders that were insensitive to GSK101. **(D)** Averaged data for the CAP-responding GSK101-insensitive cohort (*n* = 29). **(E,F)** Traces and averaged data from GSK101 + CAP responders (*n* = 27). **(G)** Summary of averaged data. ∼24% of the TRPV4-expressing RGCs responded to CAP, whereas ∼48% of TRPV1-expressing RGCs responded to GSK101. ^∗^*P* ≤ 0.05, ^∗∗^*P* ≤ 0.01, ^∗∗∗^*P* ≤ 0.001, ^∗∗∗∗^*P* ≤ 0.0001.

TRPV1 and TRPV4 channels have been implicated in optic nerve degeneration (Križaj et al., 2014; Sappington et al., 2015) and axonal neuropathies (Echaniz-Laguna et al., 2014), and shown to be sensitive to mechanical stressors (such as changes in cell volume and strain; Sudbury et al., 2010; Ryskamp et al., 2016). To categorize the relative fractions of TRPV1- and TRPV4-expressing RGCs, we used microfluorimetry. Dissociated cells were identified by the size and morphology of the somata, and responsiveness to glutamate (100 μM) and high K^+^ (35 mM; Ryskamp et al., 2011). After loading with the calcium indicator Fura-2-AM, cells were stimulated with pharmacological activators and inhibitors of TRPV1 and TRPV4 channels. The largest RGC cohort (35.08%) showed intracellular Ca^2+^ ([Ca^2+^]_i_) increases in response to the TRPV4 agonist GSK101 (25 nM) and lack of sensitivity to the TRPV1 agonist CAP (10 μM; **Figures 2A,B**). On average, in these cells, GSK101 evoked an increase in the 340/380 ratio of 0.51 ± 0.05 (*n* = 87; *P* < 0.001). As shown previously (Ryskamp et al., 2011, 2014a), the responses to the TRPV4 agonist were characterized by a transient [Ca^2+^]_i_ peak that inactivated in the continued presence of GSK101. Subsequent co-applications of the drug evinced lower-amplitude or missing Ca^2+^ responses due to tachyphylaxis (continued channel desensitization). Pretreatment with CAP did not affect the amplitude of GSK101-evoked [Ca^2+^]_i_ responses in this cohort, suggesting that tachyphylaxis is isoform-specific.

**FIGURE 3:**
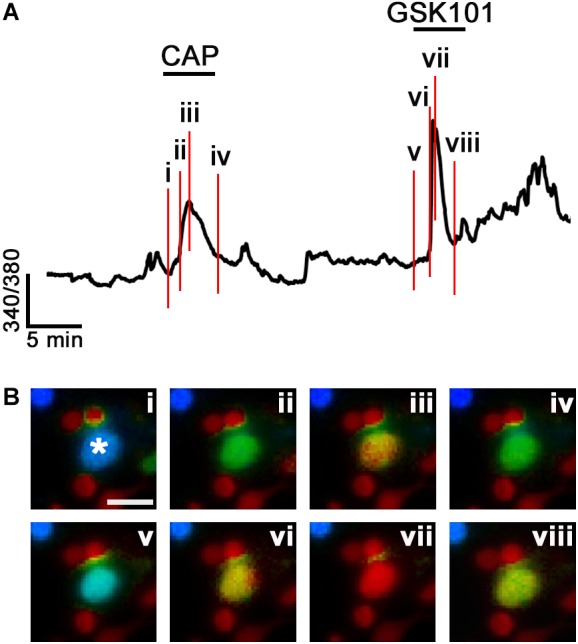
Functional coexpression of TRPV1 and TRPV4 channels in a representative RGC (*asterisk*). **(A)** The time course of the calcium response in a C57 RGC sequentially stimulated with CAP and GSK101. The red vertical lines denote the time points shown in panel **Bi–viii**. **(B)** Ratio images from the RGC (asterisk) shown in **A**. Note that [Ca^2+^]_i_ declines in the continued presence of both agonists. The small-diameter somata adjacent to the RGC are rod somata. Scale bar = 10 μm.

Another population, encompassing 11.69% of RGCs, responded to CAP administration with increased [Ca^2+^]_i_ with an average CAP-evoked ratio increase of 0.45 ± 0.07 (*n* = 29; *P* < 0.001; **Figures 2C,D**). These cells were unresponsive to GSK101, and co-application of GSK101 and CAP yielded a ratio increase of 0.39 ± 0.08 that was not significantly different from the exposure to CAP alone. The third functional type (10.89%) responded to both TRPV1 and TRPV4 agonists (0.43 ± 0.09 and 0.48 ± 0.06 ratio increases, respectively; *n* = 27; **Figures 2E,F**). A representative example of a cell classified into the third cohort is shown in **Figure 3**, with vertical lines in **Figure 3A**, corresponding to fluorescence images of free [Ca^2+^]_i_ elevations in the RGC cytosol shown in **Figure 3B**. As expected (Ryskamp et al., 2011; Jo et al., 2017), the response to both agonists desensitized in the continued presence of the agonist (**Figures 3Biv,viii**). The dataset in **Figure 2G** shows that ∼46% of total glutamate responder RGCs express TRPV4, and ∼20% express TRPV1. Another way of parsing the data shows that ∼48% of TRPV1 expressing RGCs (22.6% of total glutamate-responding cells) functionally express TRPV4 channels whereas ∼24% of TRPV4-expressing cells express TRPV1 as well.

### TRPV4 Is Coexpressed With TRPV1 in a Subset of RGCs

We next investigated whether the results from functional studies (**Figures 2**, **3**) can be mirrored by proof-of-principle histochemical evidence. TRPV4 channels can be studied with validated antibodies (Ryskamp et al., 2011; Jo et al., 2016) but the specificity of commercial TRPV1 antibodies is questionable (Gilliam and Wensel, 2011; Molnar et al., 2012). We therefore studied TRPV1Cre:Ai9 and TRPV1Cre:Ai3 retinas in which channel expression manifests in the fluorescence patterns of tdTomato and GFP reporters, respectively (Mishra and Hoon, 2010; Jo et al., 2017). Cells were evaluated in vertical sections from the central- to mid-peripheral retina in order to increase the likelihood of hitting on TRPV1-expressing RGCs (e.g., Jo et al., 2017).

**FIGURE 4:**
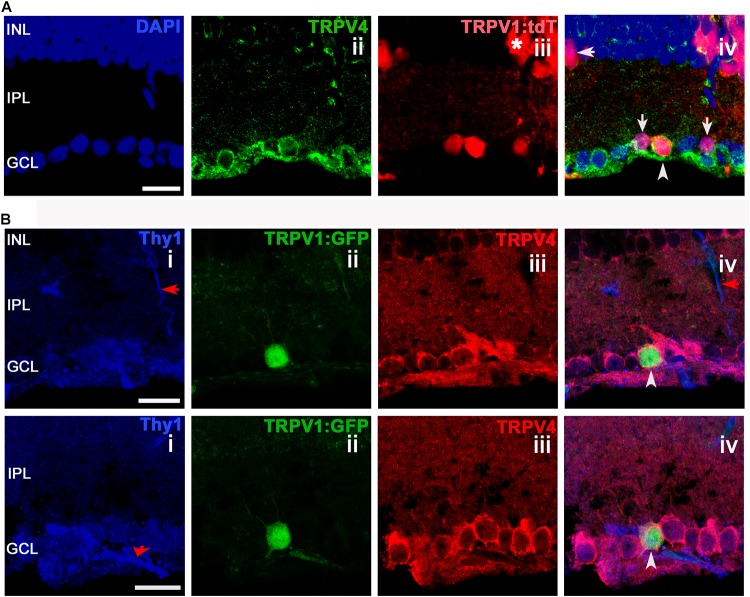
TRPV1 expression in RGCs but not amacrine cells overlaps with TRPV4-ir. Confocal microscopy, vertical sections from the mouse retina. **(A)** TRPV1Cre/Ai9 retina labeled for DAPI, TRPV4, and TRPV1. TRPV1:tdTomato is expressed in cell somata localized in ganglion cell layer (GCL), inner nuclear layer (INL), and in a subset of putative Müller cells (asterisk). RGC that colocalizes TRPV1 and TRPV4 is marked by an arrowhead in panel iv. Arrows mark putative (small-diameter) amacrine cells in RGCL and IPL that are TRPV4^-^. **(B)** TRPV1Cre/Ai3 section showing a TRPV1^+^ cell labeled by the GFP reporter. The cell was immunopositive for TRPV4 and Thy1. Red arrows donate blood vessels. Pictures were obtained from two optical sections with a thickness of 1 μm. Scale bars = 20 μm.

We found that TRPV4 is localized to a substantial population of putative RGCs (identified by Thy1 or RBPMS-ir). TRPV1^+^ cells, identified by tdT^+^ and GFP^+^ fluorescence (**Figure 4**), typically colocalized with TRPV4-ir, with rare examples (arrow in **Figure 4A**) that were immunonegative for TRPV4. Two examples of TRPV1^GFP^ cell that colocalized the RGC marker Thy1 (CD90) with TRPV4 are shown in **Figure 4B** (arrowheads). Although Thy1 labels a subset of displaced cholinergic syntaxin^+^ cells in the RGC layer (Raymond et al., 2008), the presence of TRPV4 (which is absent from amacrines; Ryskamp et al., 2011) indicates that the labeled cell is a RGC.

**FIGURE 5:**
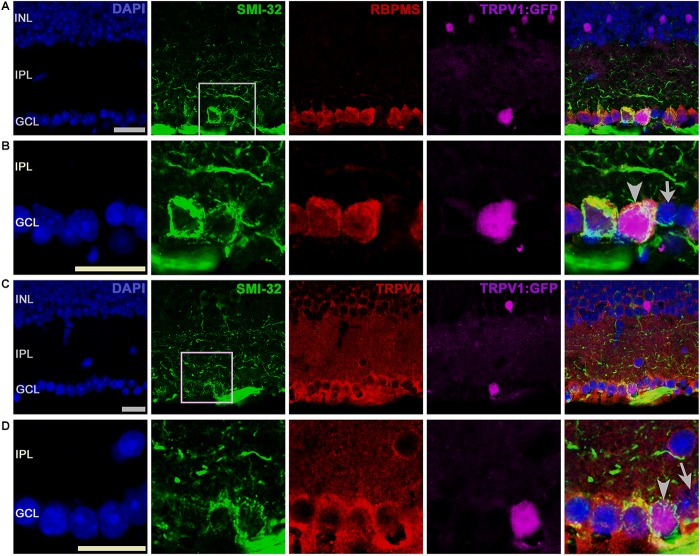
SMI-32^+^ RGCs coexpress TRPV1 and TRPV4 signals. **(A)** A representative TRPV1-expressing RGC is labeled by the GFP reporter that was immunopositive for SMI-32 and RBPMS. **(B)** Inset from **A**. **(C)** TRPV1-expressing RGCL neuron is labeled by SMI-32 and TRPV4 (**D**, arrowhead) antibodies. The arrow denotes a TRPV4- and SMI-32-immunonegative putative amacrine cell in the proximal INL. Pictures were obtained from one optical section. Scale bars = 20 μm.

We investigated whether TRPV1 and 4 channels might be expressed in SMI-32 cells, which label αRGCs, large-diameter monostratified cells that arborize in ON or OFF sublaminae of the inner plexiform layer (Coombs et al., 2006), play a role in contrast sensitivity and are sensitive to ocular hypertension (Della Santina et al., 2013; Schmidt et al., 2014; Ou et al., 2016). All TRPV1-expressing RGCL cells were immunopositive for SMI-32 and TRPV4. **Figures 5A,B** show a TRPV1-expressing (RBPMS^+^) RGC that strongly expresses SMI-32. Another example, shown in **Figures 5C,D**, showcases a TRPV1-expressing αRGC that also expresses TRPV4 (arrowhead in **Figure 5D**), whereas a TRPV1-expressing putative amacrine cell (arrow in **Figure 5C**) was TRPV4 immunonegative. We conclude that most RBPMS^+^ TRPV1^+^ cells are SMI-32^+^ and can thus be classified as αRGCs. Of SMI-32^+^ cells, 35.9% were TRPV1^+^. TRPV1 expression was detected in some Müller cells that were immunopositive for TRPV4 (asterisks in **Figure 4A**). These data suggest that RGCs sense their ambient environment through different combinations of sensory transducers, with TRPV4 channels as dominant non-epithelial vanilloid transducers. Moreover, Müller glia appear to express both vanilloid isoforms, which is consistent with their function as regulators in the retinal microenvironment (Ryskamp et al., 2015).

### TRPV4 Signaling in TRPV1-Expressing RGCs Does Not Involve Subunit Heteromerization

**FIGURE 6:**
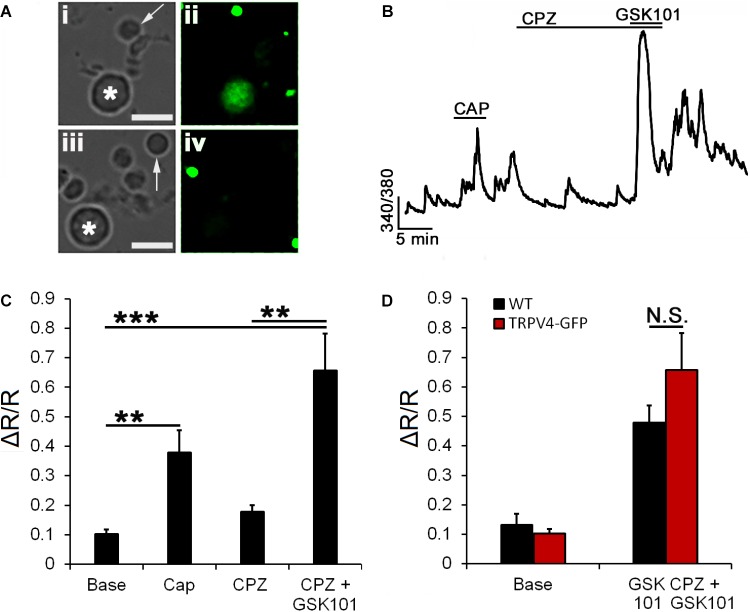
TRPV4 signaling does not require TRPV1. **(A)** Cells dissociated from transgenic TRPV4^GFP^ retinas **(i,ii)** and control retinas **(iii,iv)** show reporter expression in large-diameter neurons (asterisk) but not rod somata (arrow). **(B)** Representative trace from a TRPV4^GFP+^ RGC shows a potential response to CAP superimposed on spontaneous background Ca^2+^ transients. The TRPV1 antagonist CPZ (10 μM) did not affect the [Ca^2+^]_i_ baseline or inhibit GSK101-evoked [Ca^2+^]_i_ elevations. **(C)** Averaged data for CAP and CPZ responses in TRPV4^GFP+^ RGCs (*n* = 12). **(D)** CPZ had no effect on the amplitude of GSK101-evoked [Ca^2+^]_i_ signals (*n* = 12). Scale bar = 10 μm. N.S., *P* < 0.05, ^∗∗^*P* ≤ 0.01, ^∗∗∗^*P* ≤ 0.001.

Vanilloid TRP channels preferentially assemble into homomeric channels (Hellwig et al., 2005); however, formation of macromolecular complexes between TRPV4 and TRPC1 (Ma et al., 2015), TRPP2 (Stewart et al., 2010), and TRPV1 (Sappington et al., 2015) has been reported for endothelial, kidney, and ganglion cells, respectively. We recently reported that pharmacological blockade of TRPV4 has no effect on TRPV1-mediated calcium signals (Jo et al., 2017). Here, we took advantage of transgenic mice to test whether heteromerization with TRPV1 is obligatory for TRPV4 functionality. To test TRPV1–V4 interactions, we assessed the responsiveness to CAP in RGCs expressing a fluorescent reporter (eGFP) under the control of the TRPV4 promoter (Gu et al., 2016). Recordings from CAP responding TRPV4^eGFP+^ RGCs (**Figures 6Ai,ii**) showed an absence of effect of the TRPV1 antagonist CPZ on baseline [Ca^2+^]_i_. The amplitude of GSK101-induced [Ca^2+^]_i_ elevations in the presence of CPZ was 0.66 ± 0.12 (*n* = 12, *P* < 0.001; **Figures 6A,B**), not significantly different from GSK101 responses in WT cells (0.51 ± 0.05; **Figure 2D**). These data indicate that TRPV1 in mouse RGCs is not required for TRPV4 function and vice versa, TRPV4-mediated responses are largely unaffected by TRPV1 knockdown.

### TRPV1 Channel Does Not Influence TRPV4 Functionality in Subset of RGCs

**FIGURE 7:**
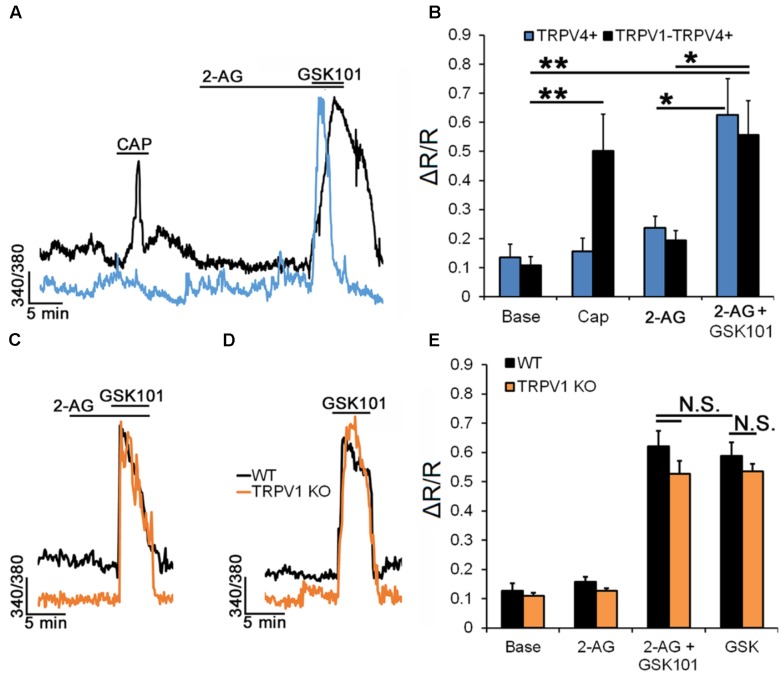
RGC TRPV4 signaling is not modulated by endocannabinoids. Recordings from TRPV4^eGFP^, wild type (C57), and TRPV1^-/-^ RGCs. **(A)** Representative traces and **(B)** cumulative averaged data for CAP and 2-AG responses in TRPV4^+^ (*n* = 10) and TRPV1-TRPV4^+^ (*n* = 10) RGCs. **(C)** Preincubation with 2-AG has no effect on the amplitude of the GSK101-evoked [Ca^2+^]_i_ response (*n* = 24). **(D)** Ablation of the *Trpv1* gene has no effect on the TRPV4 response (*n* = 8). **(E)** Cumulative averaged data from WT (black) and TRPV1 (orange) KO RGCs. Neither exposure to 2-AG nor absence of TRPV1 impact GSK101-evoked [Ca^2+^]_i_ signaling. ^∗^*P* ≤ 0.05, ^∗∗^*P* ≤ 0.01.

Expression of cannabinoid receptors in most, if not all, mammalian retinal cells (Ryskamp et al., 2014b; Jo et al., 2017) suggests that tonic and/or activity dependent release of endocannabinoids might be important for processing of visual signals. Endocannabinoids regulate TRPV1 directly and through CB1R-dependent intracellular messengers (Zygmunt et al., 1999) but were also suggested to influence TRPV4 activation (Watanabe et al., 2003; Ho et al., 2015). Exposure to the endogenous agonist of CB1 receptor 2-AG suppresses TRPV1 channels in mouse RGC (Jo et al., 2017). To establish whether 2-AG has comparable effects on RGC TRPV4 activation we recorded calcium signals from TRPV4^eGFP^ (**Figures 7A,B**), as well as wild type (black traces, **Figures 7C–E**) and TRPV1^-/-^ RGCs (orange traces, **Figures 7C–E**) in the presence or absence of 2-AG. **Figures 7B,C** show that 2-AG (1 μM) does not affect the [Ca^2+^]_RGC_ baseline and GSK101-evoked [Ca^2+^]_i_ signals in both TRPV4^+^ (0.63 ± 0.12; *n* = 10) and TRPV1-TRPV4^+^ (0.56 ± 0.12; *n* = 10) RGCs. Moreover, in TRPV1^-/-^ RGCs (**Figures 7C–E**), GSK101-evoked [Ca^2+^]_i_ signals recorded in cells preincubated with 2-AG (0.62 ± 0.05; *n* = 24) were also indistinguishable from control responses (0.59 ± 0.05; *n* = 24). Consistent with the pharmacological experiments (**Figure 6**), we found that ablation of TRPV1 has no effect on GSK101-evoked signals (*n* = 8; **Figure 7A**). These data suggest that, unlike TRPV1 signals, TRPV4 signaling in the mouse retina will resist endocannabinoid modulation.

## Discussion

This study provides a number of novel observations that are relevant for the understanding of non-canonical sensory transduction in vertebrate vision. First, we quantified the TRPV isoform expression across RGCs. This is important because it identifies potential targets for mechanical and inflammatory stressors that affect specific subpopulations of these projection neurons. Second, we determined the relative abundance and colocalization of the two most extensively studied vanilloid channel isoforms in mouse RGCs. Third, we provide evidence against obligatory functional heteromerization between TRPV1 and TRPV4 channels. Fourth, we show that a proportion of SMI-32^+^ αRGCs – cells known to be sensitive to IOP – coexpress TRPV1 and TRPV4 channels. Together, these findings suggest that projection of sensory information to retinorecipient nuclei in the midbrain is differentially distributed across subpopulations of RGCs.

Transcript analysis in mouse RGCs revealed that the cells express all four non-epithelial vanilloid mRNAs, with *Trpv4* by far the most prevalent transcript, followed by *Trpv2* and residual expression of *Trpv1* and *Trpv3* (**Figures 1A,B**). Our functional data were broadly reflective of the *Trpv* transcriptome, with ∼50% RGCs responding to the TRPV4 agonist, ∼20% responded to the TRPV1 agonist, and ∼10% responsive to agonists specific for both channels. A similar expression pattern emerged from immunohistochemical and electrophysiological analyses in the rat DRG, in which ∼89% neurons were shown to express TRPV4, ∼34% express TRPV1 channels, and 28% express projection of both channels (Cao et al., 2009) whereas TRPV1 and TRPV2 (Lewinter et al., 2004) and TRPV1 and TRPM8 displayed little coexpression (Kobayashi et al., 2005). The percentage of TRPV4-ir RGCs exceeds the size of the GSK101-responding pool, presumably because cell dissociation/separation compromises the activation of these stretch-activated channels (e.g., Ryskamp et al., 2011).

TRPV1 and TRPV4 coexpression in mouse RGCs is in accord with quantifications that had been conducted separately for each isoform (Ryskamp et al., 2011; Jo et al., 2017). Previous work localized TRPV4 to RGC somata, and primary dendrites, but there is little information about subcellular TRPV1 expression due to the lack of specificity of TRPV1 antibodies (Gilliam and Wensel, 2011; Molnar et al., 2012). Coexpression of multiple thermoTRPs isoforms in single RGCs suggests that the cells may be capable of parallel transduction of sensory information that includes osmotic gradients, mechanical strain, acidity, and biolipids such as endocannabinoids and polyunsaturated fatty acids (e.g., arachidonic acid and eicosanoids). The possibility that TRPV4 channels mediate the disproportionate sensitivity of certain neurons to mechanical stressors is supported by the observations that (i) TRPV4 activation augments excitability by stimulating TTX-sensitive currents and voltage-operated calcium channels (Li et al., 2011) and increases the firing of substantia nigra (Guatteo et al., 2005), DRG (Cao et al., 2009), hippocampal neurons (Shibasaki et al., 2007), and RGCs (Ryskamp et al., 2011), (ii) TRPV4 mutations underlie debilitating sensory and motor neuropathies (Nilius and Voets, 2013) whereas (iii) TRPV4^-/-^ mice exhibit impaired mechanical nociception (Liedtke and Friedman, 2003) and (iv) may be protected from mechanical hyperalgesia and glaucomatous neurodegeneration (Alessandri-Haber et al., 2004; Ryskamp et al., 2016). Mice lacking a functional *Trpv4* gene show impaired responses to intense mechanical stimuli but normal responses to low threshold stimulation (Liedtke and Friedman, 2003; Suzuki et al., 2003), suggesting that TRPV4 will preferentially mediate calcium signals in respond to excessive mechanical stress (for example, in hypertensive glaucoma). Consistent with this, elevated calcium levels were reported in glaucomatous RGCs (Niittykoski et al., 2010).

TRPV1 is the most extensively studied retinal vanilloid channel, with reports suggesting pre- and postsynaptic expression across multiple cell types (Yazulla, 2008; Ryskamp et al., 2014b) that include a subpopulation of RGCs (Jo et al., 2017). Although *Trpv1* mRNA levels in RGCS were negligible compared to *Trpv2* and *Trpv4* expression, CAP-responding cells constituted ∼20% of the overall magnetoseparated population. This suggests that low expression of the *Trpv1* gene is sufficient to support TRPV1-mediated Ca^2+^ entry in a substantial mouse RGC cohort. Our findings in the retina mirror previous studies the brain, which was reported to show low *Trpv1* expression (Cavanaugh et al., 2011) even though neurons across multiple brain regions respond to CAP with TRPV1-dependent modulation of synaptic plasticity and vesicle release (Gibson et al., 2008; Wu et al., 2014; Fenwick et al., 2017). While physiological functions of retinal TRPV1 have not been clearly defined, its localization to αRGCs, which respond to modest IOP elevations with dendritic and synaptic remodeling (Ou et al., 2016) potentially links pressure-dependent RGC excitability (Weitlauf et al., 2014) and apoptosis (Sappington et al., 2009) to the early loss of large-diameter RGCs exposed to glaucomatous stressors such as IOP (Glovinsky et al., 1991). Arguing against a direct TRPV1 role in pressure transduction are the limited expression of the channel in RGCs and the reports that TRPV1 ablation augments RGC injury in a mouse model of ocular hypertension (Ward et al., 2014). There is also conflicting evidence about whether TRPV1 is expressed in retinal glia, as the channel was reported in rabbit but not detected in rat Müller cells (Leonelli et al., 2009; Martínez-García et al., 2013). Our analysis of reporter mice shows that TRPV1 is expressed in a subset of Müller glia but it remains unclear whether this was due to non-uniformity of *Trpv1* expression or an artifact resulting from differential expression of the reporter transgene.

This is the first report that a small but significant fraction of RGCs (∼10%) functionally coexpress TRPV1 and TRPV4 isoforms. Interestingly, this cohort included cells that were immunopositive for SMI-32, a marker of αRGCs which form four independent mosaics within the IPL-RGCL (Krieger et al., 2017), include M4 ipRGCs (Schmidt et al., 2014), and constitute one of the fastest pathways for retina–brain information transfer. Whether native TRPV1 and TRPV4 channels are capable of heteromultimerization has been controversial given that subunit interactions predicted by FRET studies (Cheng et al., 2007) and co-immunoprecipitation (Sappington et al., 2015) have not been not substantiated by investigations of TRPV1-4 subunit expression in heterologously expressing cells (Hellwig et al., 2005). TRPV4^-/-^ mice exhibit impaired mechanical nociception but show conserved TRPV1-mediated responses to noxious heat (Liedtke and Friedman, 2003; Suzuki et al., 2003). We expand on these studies to show that heteromerization is not obligatory for the activation of native neuronal TRPV1 or TRPV4 channels as indicated the observation that neither activation nor inhibition of TRPV1 affect the amplitude and kinetics of GSK101-evoked calcium responses. *Vice versa*, pretreatment with TRPV4 agonists/antagonists had no effect on the cells’ responsiveness to CAP. We also found that (i) desensitization of one isoform has little effect on the agonist-evoked responsiveness of the other and (ii) CB1 receptor activation which inhibits TRPV1 channels (Jo et al., 2017) has no effect on TRPV4-mediated signals in TRPV1-expressing RGCs; this finding mirrors the analyses in DRG neurons which showed that native TRPV1 and TRPV4 currents can be explained by single channel properties of each channel (Premkumar et al., 2002; Kim et al., 2016). It is possible that interactions between the two channels will emerge under pathological circumstances, as either channel can contribute to mechanical hyperalgesia (Vennekens et al., 2008; Huynh et al., 2014) and both were linked to optic neuropathy (Ryskamp et al., 2011; Weitlauf et al., 2014). Indeed, TRPV1 activation that is not harmful in healthy tissue can be pathological during mechanical hyperalgesia and facilitated by pro-inflammatory molecules that have been implicated in glaucoma such as ATP, prostaglandins, and arachidonic acid metabolites (Nilius and Szallasi, 2014). We hypothesize that TRPV1/4 sensitization (“allodynia”) amplifies pressure-induced neuronal damage through eicosanoid products of CYP450, which activate TRPV4 (5′6′-EET, 11′,12′-EET) and TRPV1 (12-(S)-HETE, 20-HETE) (Watanabe et al., 2003; Wen et al., 2012; Ryskamp et al., 2014b) or N-arachidonoyl taurine which activates both channels (Bradshaw et al., 2013). TRPV1/4-dependent mechanical allodynia is a characteristic feature of neurogenic inflammatory and neuropathic pain paradigms in sensory neurons (Alessandri-Haber et al., 2004; Amadesi et al., 2006; Grant et al., 2007). Importantly, hyperalgesia-associated properties of thermoTRPs channels would also augment the susceptibility of retinal neurons and glia to glaucomatous injury (Križaj, 2016). Among inflammatory agents that sensitize TRPV1 and TRPV4 are ATP, bradykinin, prostaglandin E2, and PAR2 agonists which have been linked to cytotoxicity and neurodegeneration (Grant et al., 2007; Nilius and Szallasi, 2014; Lu et al., 2015). However, certain messenger molecules may preferentially stimulate one isoform over the other. For example, TRPV4 signaling in RGCs is unaffected by the long-chain unsaturated acyl-amide 2-AG that potently modulates TRPV1 activation (Bradshaw et al., 2013; Ryskamp et al., 2014b; Jo et al., 2017).

Collectively, these findings extend our understanding of how non-canonical sensory stimuli are transduced in mammalian RGCs. We know that the devastating effects of pressure, strain, swelling, ocular trauma and inflammatory inputs on vision tend to be associated with early effects of mechanical stress on the viability and function of RGCs (Križaj, 2016). It remains to be seen whether synergistic activation of TRPV1 and 4 under pathological conditions unlocks novel models of sensory transduction, as observed for somatosensory afferents, in which combined expression of TRPV1, TRPM3, and TRPA1 is required for the transduction of noxious heat (Vandewauw et al., 2018). A non-mutually exclusive possibility is that the two vanilloid isoforms impart complementary sensory information that could be important for signaling in specific RGC classes such as αRGCs. Because mouse RGC subpopulations tend to be conserved in primates (Chalupa and Williams, 2008), our findings might extend across phylogenetic domains.

## Author Contributions

ML and DK conceived the project. JB, DY, AJ, and ML performed the experiments. HH contributed transgenic mice. ML and DK analyzed the data and wrote the paper.

## Conflict of Interest Statement

The authors declare that the research was conducted in the absence of any commercial or financial relationships that could be construed as a potential conflict of interest.
